# Identification of SARS-CoV-2 Cell Entry Inhibitors by Drug Repurposing Using *in silico* Structure-Based Virtual Screening Approach

**DOI:** 10.3389/fimmu.2020.01664

**Published:** 2020-07-10

**Authors:** Shweta Choudhary, Yashpal S. Malik, Shailly Tomar

**Affiliations:** ^1^Department of Biotechnology, Indian Institute of Technology Roorkee, Roorkee, India; ^2^Division of Biological Standardization, Indian Veterinary Research Institute, Bareilly, India

**Keywords:** RNA viruses, SARS-CoV-2, receptor-binding domain, ACE2, COVID-19, drug-repurposing

## Abstract

The rapidly spreading, highly contagious and pathogenic SARS-coronavirus 2 (SARS-CoV-2) associated Coronavirus Disease 2019 (COVID-19) has been declared as a pandemic by the World Health Organization (WHO). The novel 2019 SARS-CoV-2 enters the host cell by binding of the viral surface spike glycoprotein (S-protein) to cellular angiotensin converting enzyme 2 (ACE2) receptor. The virus specific molecular interaction with the host cell represents a promising therapeutic target for identifying SARS-CoV-2 antiviral drugs. The repurposing of drugs can provide a rapid and potential cure toward exponentially expanding COVID-19. Thereto, high throughput virtual screening approach was used to investigate FDA approved LOPAC library drugs against both the receptor binding domain of spike protein (S-RBD) and ACE2 host cell receptor. Primary screening identified a few promising molecules for both the targets, which were further analyzed in details by their binding energy, binding modes through molecular docking, dynamics and simulations. Evidently, GR 127935 hydrochloride hydrate, GNF-5, RS504393, TNP, and eptifibatide acetate were found binding to virus binding motifs of ACE2 receptor. Additionally, KT203, BMS195614, KT185, RS504393, and GSK1838705A were identified to bind at the receptor binding site on the viral S-protein. These identified molecules may effectively assist in controlling the rapid spread of SARS-CoV-2 by not only potentially inhibiting the virus at entry step but are also hypothesized to act as anti-inflammatory agents, which could impart relief in lung inflammation. Timely identification and determination of an effective drug to combat and tranquilize the COVID-19 global crisis is the utmost need of hour. Further, prompt *in vivo* testing to validate the anti-SARS-CoV-2 inhibition efficiency by these molecules could save lives is justified.

## Introduction

The world is facing a dire situation of global public health emergency due to a viral pandemic of severe febrile pneumonia like respiratory syndrome caused by a novel coronavirus, named SARS-CoV-2, causing the COVID-19 disease. SARS-CoV-2, a member of the *Coronaviridae* family, is a type of positive-sense, single-stranded enveloped RNA virus responsible for causing infections in avian, mammalian, and marine species across the world (1, 2). Clinical onset of infection in COVID-19 is characterized by symptoms as headache, dry cough, and fever; in severe cases multi-organ failure, and even deaths (3). As of April 13th 2020, the outbreak has adversely affected more than 1,800,000 people globally, and about 100,000 deaths have already been reported from Mainland China and rest of the 213 affected countries (4).

Infections caused by alpha-coronaviruses (NL63-CoV and HCoV-229E) are usually mild and asymptomatic, whereas beta-coronaviruses like severe acute respiratory syndrome coronavirus (SARS-CoV) and Middle East respiratory syndrome coronavirus (MERS-CoV), have caused serious epidemics (5). In the year 2002, SARS-CoV emerged as an epidemic in China and resulted in ~8,000 reported cases (6). Recurrence in the form of MERS-CoV was later reported in Saudi Arabia, with a fatality rate of 35% (7, 8). NL63-CoV, HCoV-OC43, and HCoV-HKU1 are a few other coronaviruses responsible for causing infections in humans (9).

Re-emergence of coronaviruses, as SARS-CoV-2 in the end of year 2019, has put the world on high alert and has created an alarming situation demanding an urgent treatment to preclude the potential death of infected patients (2, 10). Despite extensive efforts worldwide by researchers, there are still no effective antiviral drugs or therapies available that could treat patients or prevent the virus transmission. Current prevention and treatment efforts are directed on quarantine and containment of infected patients to prevent human to human transmission (10, 11). However, reports are available on repurposing the antiviral drugs like remdesivir, lopinavir, ritonavir, and anti-malarial drug chloroquine against SARS-CoV-2 (12). Additionally, neutralizing monoclonal antibody-based therapeutics are also being developed to combat COVID-19 crisis (13, 14).

Coronavirus infection in humans is driven mainly by interactions between envelope-anchored spike glycoprotein (S-protein) of coronavirus and the host cell receptor, angiotensin-converting enzyme 2 (ACE2) (15, 16). The S-protein is made up of two subunits, S1 as the receptor-binding domain (RBD) and S2 subunit is responsible for the fusion of viral membrane and the host cellular membrane (17). S2 subunit of SARS-CoV-2 is highly conserved with ~99% similarity whereas the S1 subunit shares 70% similarity with other bat SARS-CoV and human SARS-CoV, but the core RBD domain is highly conserved among them (2, 18). Furthermore, the residues of S-RBD of SARS-CoV-2 are highly conserved when compared to SARS-CoVs from bats, human, and palm civet cat. The affinity between S-RBD of SARS-CoV-2 and ACE2 is found to be approximately ten times higher when compared with SARS-CoV RBD (year 2003), implying that ACE2 is the specific receptor which is responsible for the binding of virus to the host cell membrane (8, 19). Evidently, the key residues of SARS-CoV RBD (Tyr442, Leu472, Asn479, Asp480, and Thr487) are hypothesized to have undergone natural selection in SARS-CoV-2 and have been proposed to play a critical role in cross-species transmission of coronaviruses (19). Based on previous studies, Lys31 and Lys353 located on ACE2 are considered to be virus-binding hotspot residues liable for binding of S-protein (1, 20). In human ACE2 receptor, hotspot 31(Lys31) is made up of salt bridge between Lys31 and Glu35, and hotspot 353 is made up of another salt bridge between Lys353 and Asp38, surrounded by a hydrophobic environment (20). SARS-CoV-2 recognizes human ACE2 by its residues Gln493 and Leu455, which are proposed to form favorable molecular interactions with hotspot 31, thereby enhancing viral binding to human ACE2. Additionally other key residues of S-protein provide more support for hotspot 31(SARS-CoV-2: Leu455, Phe486, Ser494; SARS-CoV: Tyr442, Leu472, and Asp480). In SARS-CoV-2, residue 494 which is a serine also strengthens structural stability of hotspot 353 (Lys353) of ACE2 receptor (1).

Intriguingly, detailed molecular analysis and characterization of these interactions between ACE2 receptor and S-RBD of SARS-CoV-2 are essential to develop vaccines or therapeutic drugs for prevention and treatment of infections SARS-CoV-2. Computational screening of large compound libraries can be done against SARS-CoV-2 targets, based on epitopes, polyprotein, S-RBD domain of SARS-CoV-2, or for the virus receptor ACE2. Repurposing them for coronavirus infections can be an alternative approach that could help to discover potential antiviral molecules rather quickly (21). To this end, structure-based virtual screening approach was used for identifying inhibitor molecules targeting SARS-CoV-2 virus-host cell interaction, using the crystal structure of ACE2 complexed with S-RBD and the newly released whole genome sequence of SARS-CoV-2 (22, 23). Given that ACE2 is the key receptor for S-RBD, the hotspot 31 and hotspot 353 residues were targeted in this study, to identify small molecules that could help in preventing SARS-CoV-2 infections. This framework was reiteratively applied to identify small molecules targeting both the virus binding hotspot 31 and hotspot 353 on ACE2 receptor, and the residues of S-RBD of SARS-CoV-2. Binding interactions of potential antiviral molecules identified in this study, were validated using *in silico* structure-based molecular docking and simulation approach. This study has identified potential anti-SARS-CoV-2 agents, which can be directly tested for *in vitro* and *in vivo* studies, to combat a global threat of COVID-19.

## Materials and Methods

### Hardware and Software

All computational study work was done on macOS Mojave workstation with 8-core Intel Xeon E5 processor. MD simulation studies were performed on LINUX workstation using GROMOS96 43a1 force field in GROMACS 5.1.1 suite. Bioinformatics software, such as PyRx 0.8 (24), Open Babel (25), AutoDock Vina (26), PyMol (27), GROMACS (28) and online resources like SWISS MODEL (29), HADDOCK (30), RCSB PDB (31), NCBI (32), ProCheck at RCSB validation server (33), ProSA-web (34), SAVES-Verify3D server (35), etc. were used in this study.

### 3D Homology Model Generation of S1-Subunit

Homology modeling for S1-subunit of S-protein (residues 319-529) of SARS-CoV-2 was done using SWISS-MODEL. NCBI was used to obtain target sequence for SARS-CoV-2 based on whole genome sequence of SARS-CoV-2 (GenBank accession number: MN908947.3). Crystal structure of SARS-CoV-2 S-protein (PDB ID: 6VSB) was the template hit obtained which has a sequence identity of ~99%. This was used as a template to build three-dimensional model of S-RBD protein of SARS-CoV-2. Quality assessment of the predicted three dimensional (3D) homology model of S-RBD protein was done using PROCHECK, followed by validation using ProSA plot, SAVES server, and Verify 3D. The best-mapped model with the least number of residues in the disallowed region was selected and used for the virtual screening to identify compounds that bind S-RBD.

### Choice of Ligand Library

For structure-based repurposing of clinically approved drugs, LOPAC drug library (Library of Pharmacologically Active Compounds, Sigma-Aldrich,St. Louis, MO) of ~1,280 molecules, was used for screening to find potential antiviral drugs or compounds. LOPAC library contains marketed drugs as well as pharmacologically active compounds that possess well-characterized activities. These potential drug molecules were docked into crystal structures of ACE2 and modeled S-RBD of SARS-CoV-2.

### Structure-Based Virtual Screening Against ACE2 Receptor and S-RBD

For this study, crystal structure of ACE2 receptor protein (PDB ID: 2AJF) and the spike protein S-RBD, which has been modeled using template of S-protein of SARS-CoV-2 (PDB ID: 6VSB), was used. The three-dimensional structures of drugs or small chemical molecules retrieved from LOPAC library were of SDF type. Open Babel software was used to convert all ligands into PDBQT type. AutoDock Vina (Version 4.2) and PyRx were used to screen FDA approved LOPAC library molecules centering around hotspot 31 and hotspot 353 residues of ACE2 protein of the host cell. Additionally, modeled structure of S-RBD of SARS-CoV-2 was also used for *in silico* screening of therapeutic molecules from LOPAC library, targeting important residues (Leu455, Phe486, Asn487, Gln493, and Ser494), responsible for recognizing hotspot 31 and hotspot 353 of ACE2 receptor. Top hit compounds, targeting specific residues of ACE2 and S-RBD, were selected and further analyzed by AutoDock Vina for identifying specific interactions involved in binding of molecules to the targets.

### Molecular Docking

Molecular docking studies of selected compounds into protein targets were carried out using AutoDock Vina. Two different sets of docking studies were conducted- one set for modeled S-RBD of SARS-CoV-2 and the other set for ACE2 protein of the host cell. For both studies, proteins were pre-processed by removal of all water and addition of kollman charges. Hydrogen bond (H-bond) optimization was done and Gasteiger charges were added to it using AutoDock MGL tools 1.5.6. A receptor grid-box was generated by AutoGrid4 with grid box dimensions of 60 Å × 80 Å × 60 Å with spacing of 0.447 Å centering around hotspot residues Lys31, Glu35, Asp38, and Lys353 for ACE2 protein. Grid box for S-RBD was also set with spacing of 0.442 Å and dimensions of 62 Å × 82 Å × 82 Å centering around residues Leu455, Phe486, Asn487, Gln493, and Ser494. Lamarckian Genetic Algorithm (GA) in combination of grid based energy evaluation method was used for docking. The program was run for a total number of 50 Genetic algorithm runs. Other parameters were set as default and the final result obtained was analyzed manually by PyMol and LigPlot.

### Molecular Dynamics Simulation

Both ACE2 protein and S-RBD protein, and their respective screened compounds were subjected to molecular dynamics (MD) simulation studies to assess the flexibility and stability of protein-ligand interactions. For this purpose, GROMACS 5.4.1 suite was used to carry out all simulation studies using GROMOS96 43a1 force field on a LINUX-based workstation. Ligand parameters and topology files were generated using PRODRG server. Furthermore, for solvation, ions, and water molecules were added to neutralize whole cubic system. Using steepest descent method, energy minimization step was performed followed by equilibration of constant number of particles, volume, and temperature (NVT), constant number of particles, pressure, and temperature (NPT). NVT equilibration was done at 300K with short range electrostatic cut-off of 1.2 nm and regulation of temperature was done by using Berendsen temperature coupling method. Further, the next phase of equilibration NPT was performed and coordinates were generated at every 1 ps. Finally, 50 ns MD production run was performed with an integration time frame of 2fs and the trajectories were generated after every 10 ps. The conformations generated during the production step were used for calculating RMSD values of protein-ligand complexes.

## Results

### Identification of ACE2 Receptor Binding Molecules

To mediate entry inside host cell, the trimeric S-glycoprotein of coronavirus binds to the host cell surface receptor ACE2 via S-RBD of S-protein (36). ACE2 is a membrane glycoprotein containing a claw like N-terminal peptidase domain made up of α-helical lobes present on outer surface, responsible for interacting with bowl-shaped cavity on S-RBD (20). In the sequence of SARS-CoV-2, the S-RBD residues directly interacting with ACE2 receptor, are similar to that of SARS-CoV, strongly signifying that ACE2 is playing a central role in SARS-CoV-2 entry into host-cell (36, 37). Lys31 and Lys353 are reported to be the two main hotspot virus-binding sites located on ACE2 at the virus-receptor interface for NL63-CoV and SARS-CoV (1, 20). Recent published data suggests that hotspot 31 is made up of salt bridge between Lys31 and Glu35, and hotspot 353 comprises of a salt bridge between Lys353 and Asp38, both buried in hydrophobic environment (1, 20).

**Figure 1:**
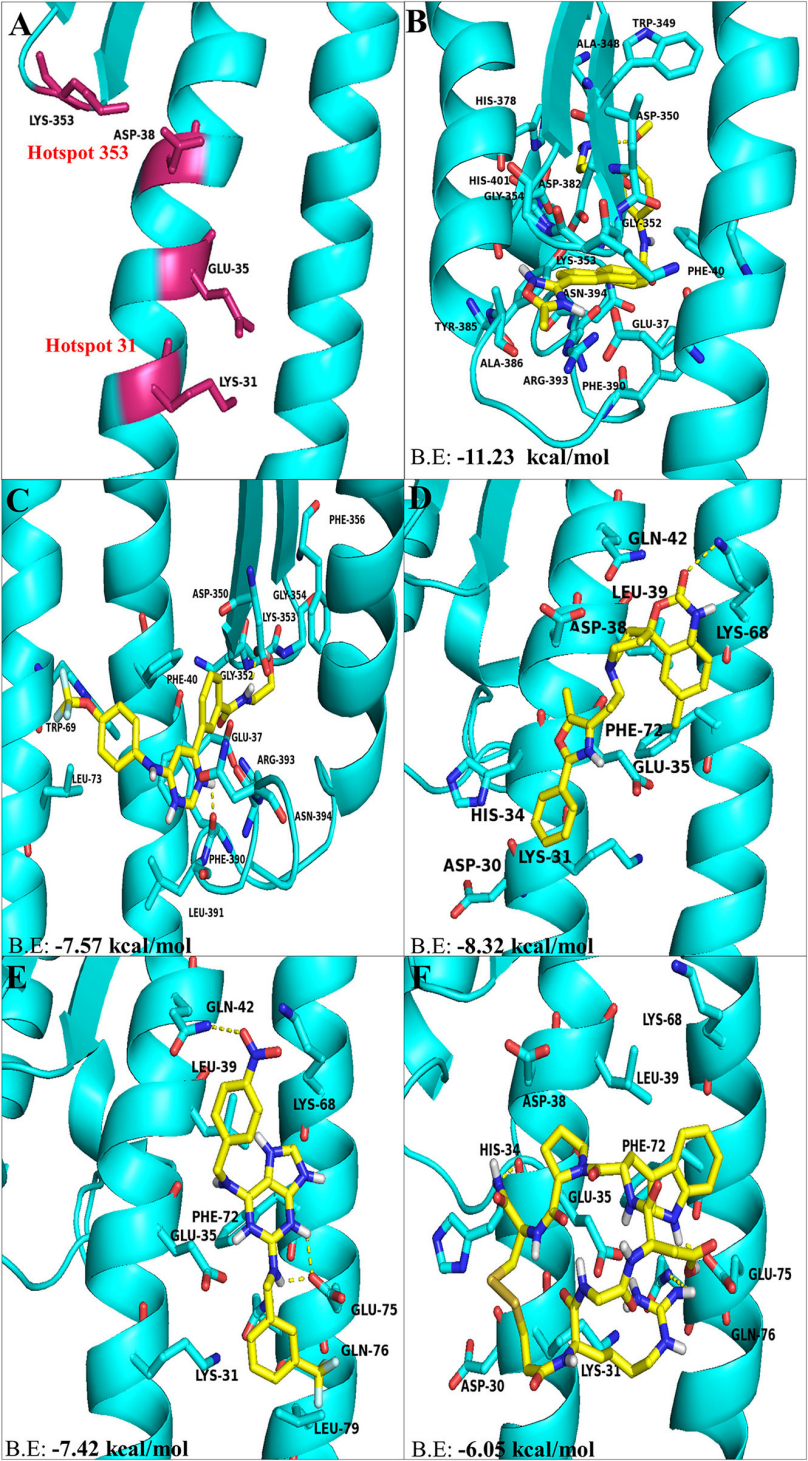
The top hit selected ligands from LOPAC library showing molecular interactions with ACE2 receptor of the host cell. **(A)** Hotspot 31 and hotspot 353 residues of ACE2 receptor responsible for recognizing S-RBD of SARS-CoV-2. **(B)** Molecular interactions of GR hydrochloride with ACE2 receptor. **(C)** Molecular interactions of GNF-5 with ACE2 receptor. **(D)** Molecular interactions of RS504393 with ACE2 receptor. **(E)** Molecular interactions of TNP with ACE2 receptor. **(F)** Molecular interactions of eptifibatide acetate with ACE2 receptor. Blue ribbons corresponds to residues of ACE2 receptor and yellow stick model represents residues of Ligands. BE, Binding energy.

Therefore, the virus binding hotspots on ACE2 receptor were targeted to identify molecules from FDA approved LOPAC library, which is expected to block ACE2 receptor and its interactions with the virus. Computer based high throughput screening was done using PyRx and AutoDock Vina with a grid box centering on Lys31 and Lys353 hotspot residues (Figure 1A). The top hit ligand candidates were scored based on their binding energies for ACE2 protein. Best 5 molecules were selected on the basis of RMSD values, molecular interactions with interface residues and binding energies. GR 127935 hydrochloride hydrate, GNF-5, RS504393, TNP and Eptifibatide acetate were the top hit compounds obtained, which targeted ACE2 host-virus interface (Figures 1B–F). To gain further insights into the interactions present at ligand-ACE2 interface, each of the selected molecule was docked into ACE2 protein using AutoDock Vina. Top scoring ligands based on their binding affinities and visual analysis of docked complexes for their capability to form H-bond and other interactions with ACE2 virus-binding motifs are documented in Table 1.

**Table 1:** Binding energies, polar and hydrophobic interaction of selected compounds screened against ACE2 receptor of host cell.

**Ligand**	**Binding energy (kcal/mol)**	**Interactions**		
		**H-Bonds**	**Bond length(Å)**	**Hydrophobic interactions**
**GR hydrochloride**	**−11.23**	N5-OE2(Glu37) O2-N(Asp350)	2.87 2.95	Phe40, Ala348, Trp349, Gly352, Lys353,Gly354, His378, Asp382, Tyr385, Ala386, Phe390, Arg393, Asn394, His401
**RS504393**	**−8.32**	O2-NZ(Lys68)	2.79	Asp30, Lys31, His34, Glu35, Asp38, Leu39, Gln42, Phe72
**TNP**	**−7.42**	O2-NE2(Gln42)	2.94	Lys31, Glu35, Leu39, Lys68, Phe72, Gln76, Leu79
		N2-OE2 and N5-OE2(Glu75)	2.92 and 2.60	
**GNF-5**	**−7.57**	O3-NE1(Trp69)	2.83	Glu37, Phe40, Leu73, Lys353, Gly354, Phe356, Leu391, Asn394
		O2-OD1 and N3-OD1(Asp350)	2.57 and 2.82	
		O2-N(Gly352)	3.08	
		O-N1(Phe390)	2.64	
		O1-NH1(Arg393)	2.75	
**Eptifibatide acetate**	**−6.05**	O-N10(His34)	2.88	Lys31, Glu35, Asp38, Leu39, Lys68, Phe72
		N11-OE2(Glu75)	2.75	
		N9-NE2(Gln76)	3.27	

*Bold values represents names of ligands and their respective binding energies*.

### Comparison of Molecular Interactions Between ACE2 Receptor and Ligands

**Figure 2:**
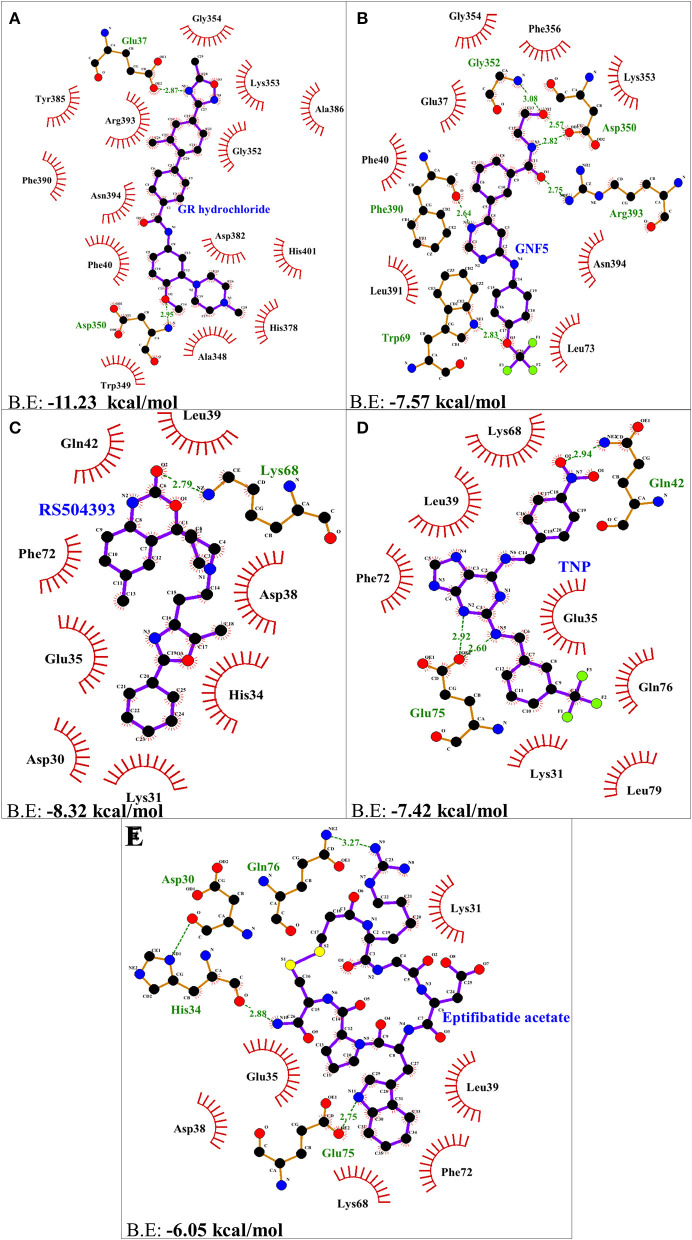
Two dimensional representation of H-bonds and hydrophobic interactions of selected compound with ACE2 receptor using LigPlot. **(A)** GR hydrochloride **(B)** GNF-5 **(C)** RS5049393 **(D)** TNP **(E)** Eptifibatide acetate. Ligands are colored and represented in purple color, H-bonds are displayed in green dotted lines, red stellations represents hydrophobic interactions, and bonds of proteins are shown in brown color.

Molecular docking using AutoDock Vina, for the top 5 molecules of the LOPAC library obtained by screening were analyzed by PyMol and LigPlot. GR 127935 hydrochloride hydrate (GR hydrochloride) displayed highest binding energy (−11.23 kcal/mol), makes 2 H-bonds with ACE2 receptor (Figure 2A). Apart from these, hydrophobic interactions are also observed including hotspot residue Lys353 and other adjacent residues like Phe40, Ala348, Trp349, Gly352, Gly354, His378, Asp382, Tyr385, Ala386, Phe390, Arg393, Asn394, and His401 clearly depicting its ability to bind and block interactions with residues of hotspot 353 (Figures 1B, 2A). Ligand GNF-5 (B.E= −7.57 kcal/mol) interacts with Lys353 through hydrophobic bond (Figure 1C). GNF-5 possessed maximum numbers of hydrogen bonds involving Trp69, Asp350, Gly352, Phe390, and Arg393 residues along with hydrophobic interactions, displaying its affinity toward hotspot 353 (Figures 1C, 2B). Key hydrophobic interactions playing a significant role for these ligands involve Phe40, Lys353, Gly354, and Asn394 along with other residues (Figures 1B,C, 2A,B). These interactions clearly demonstrate that GR hydrochloride and GNF-5 are compounds that could potentially inhibit virus, binding to hotspot 353 (Table 1). Docked conformations of ligand RS504393 (B.E= −8.32 kcal/mol), TNP (B.E= −7.42 kcal/mol), and Eptifibatide acetate (B.E= −6.05 kcal/mol) suggests that these ligands are displaying affinity toward residues of hotspot 31, and to some extent toward hotspot 353 also, showing hydrophobic interaction with Asp38 (Figures 1D–F). TNP interacts with ACE2 forming 3H-bonds with Gln42 and Glu75 whereas RS504393 interacts with ACE2 with H-bonding with Lys68. Hydrophobic interactions reported here for TNP are Lys31, Glu35, Leu39, Lys68, Phe72, Gln76, and Leu79. RS504393 interacts with Asp30, Lys31, His34, Glu35, Asp38, Leu39, Gln42, and Phe72 through hydrophobic interactions as shown in Table 1 (Figures 2C,D). Eptifibatide acetate interacts with ACE2 through 3H-bonds with residues His34, Glu75 and Gln76, and hydrophobic bonds with Lys31, Glu35, Asp38, Leu39, Lys68 and Phe72, displaying its greater affinity toward hotspot 31 (Figure 2E). MD simulation was performed to check the stability of selected compounds with ACE2 receptor protein. RMSD curves for all protein-ligand complexes attained equilibrium after 20 ns and fluctuations were found to be in the range of 0.25 to 0.31 nm for GR hydrochloride, RS504393, TNP and Eptifibatide acetate, and 0.35 to 0.4 nm for GNF-5, depicting that binding of molecules to ACE2 protein resulted in formation of stable complexes (Figures S1A–E). Given the results from all set of dockings, our study provides evidence that these identified molecules interacting with hotspot 31 and hotspot 353 specifically, if repurposed would prove to be potential drugs for further studies.

### Structure of S-RBD of SARS-CoV-2

The key determinant of host specificity of coronavirus is the surface anchored S-protein responsible for recognizing host cell receptor ACE2 through its S1 subunit. The central residues of S1 (NL63-CoV: 481-615; SARS-CoV: 306-527) are reported to contain the receptor binding domain (RBD), responsible for high affinity binding to ACE2 receptor (20). Because of sequence similarities between RBD of SARS-CoV-2 and SARS-CoV, it is hypothesized that SARS-CoV-2 infects the host cell via ACE2 receptor through binding of its RBD region of the S-protein (8).

**Figure 3:**
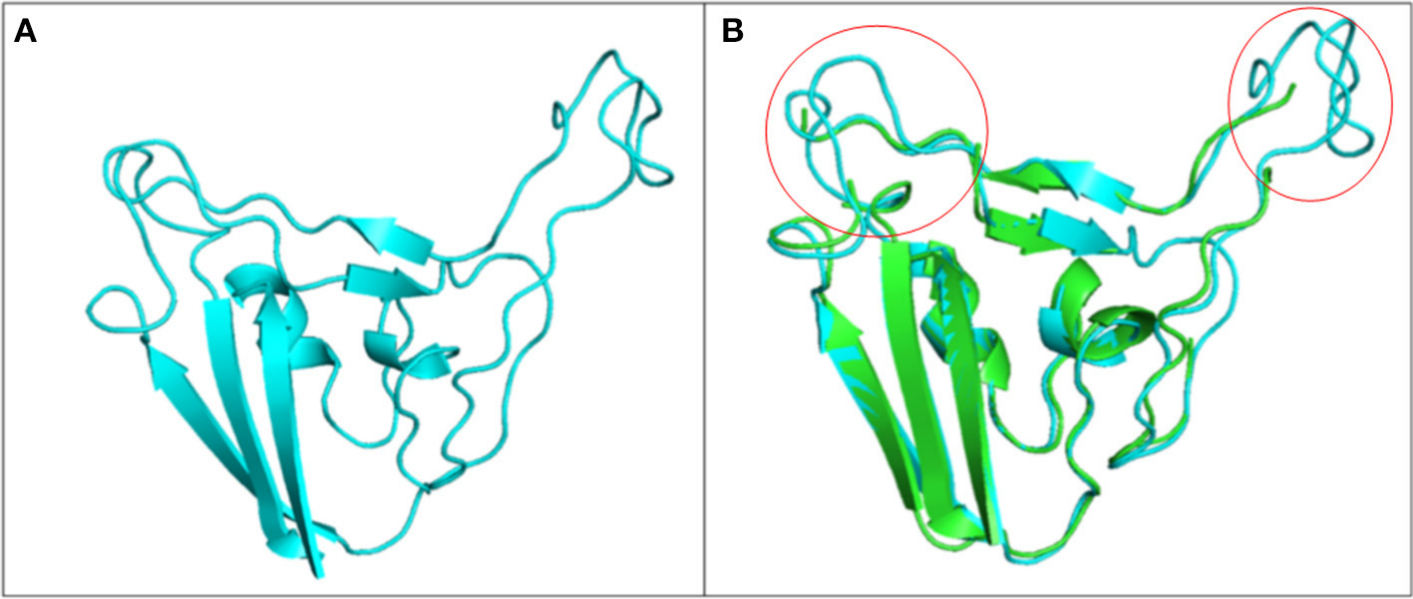
Structure of S1 subunit of SARS-CoV-2 (PMDB ID: PM0082972). **(A)** Cartoon representation of predicted S1 subunit of SARS-CoV-2. **(B)** Superimposition of template (PDB ID: 6VSB) and modeled S-RBD of S-protein. Predicted S-RBD and template are sky blue and green in color. Encircled area represents missing residues in loops of template S-protein which are modeled for S-RBD protein of SARS-CoV-2 using SWISS MODEL.

**Figure 4:**
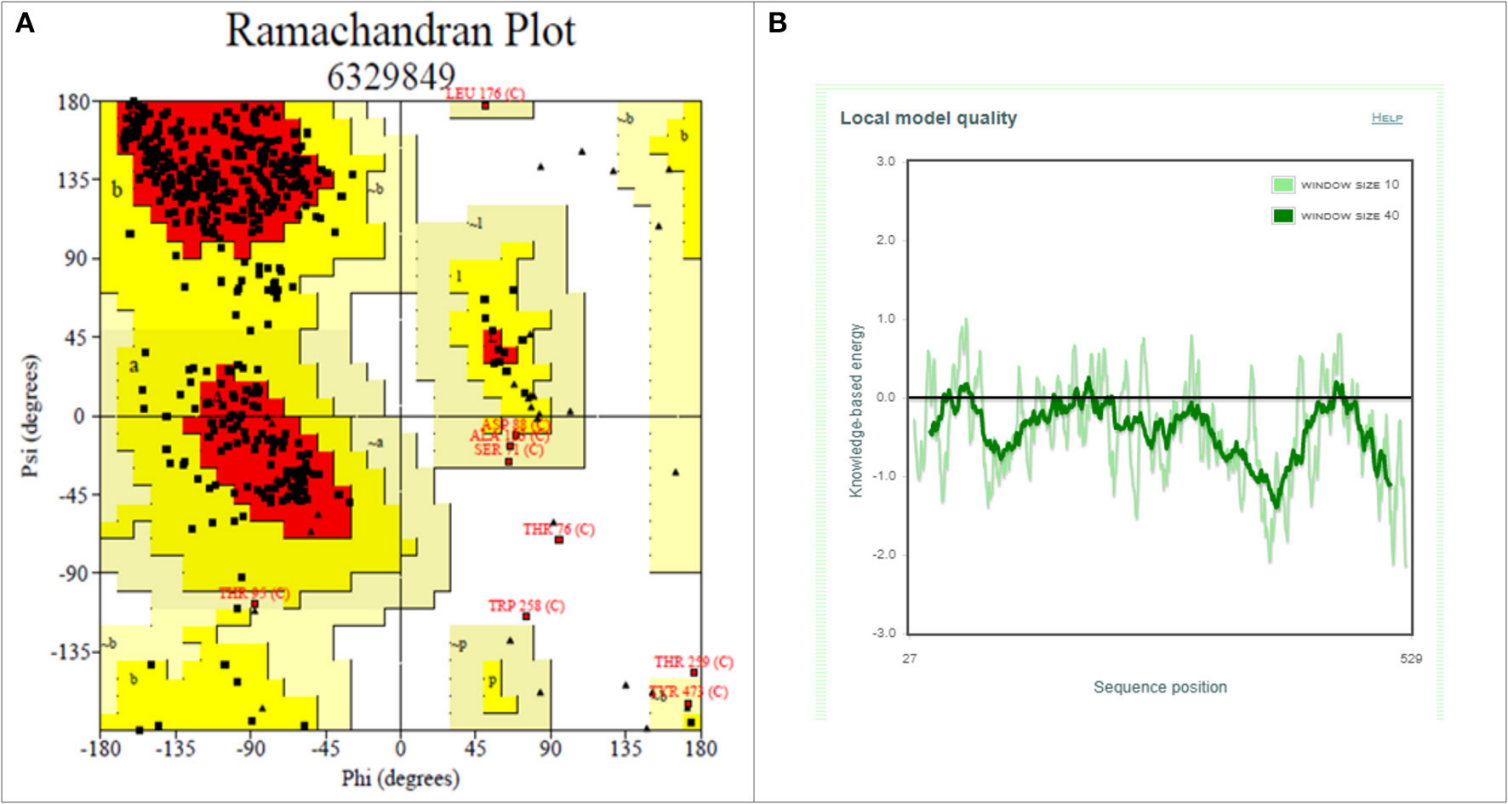
Structure validation of S1 subunit of S-protein by ProCheck and ProSA server. **(A)** ProCheck Ramachandran Plot where red, bright yellow and light yellow color represents that 99.4% residues of predicted S1 subunit of S-protein are present in favorably allowed region and 0.7% residues are present in disallowed region (lightest yellow). **(B)** Energy profile of modeled spike protein of SARS-CoV-2 as calculated by ProSA.

Drug molecules targeting the S-protein has the potential to cure COVID-19 infections and to tackle the pandemic. In this study, S1 subunit of SARS-CoV-2 was targeted by *in silico* approach to repurpose drug molecule that binds the S-RBD and blocks its interaction with ACE2 receptor, rendering it incapable to infect host cell. Since the newly published structure of SARS-CoV-2 S-protein (PDB ID: 6VSB) lacks important loop residues of S-RBD domain proposed to be involved in receptor binding, therefore a homology model was generated utilizing it as a template (Figures 3A,B). A 3D model of S1 subunit of SARS-CoV-2 S-protein, was predicted using SWISS MODEL (NCBI reference sequence: MN908947.3) and the pre-fusion structure of SARS-CoV-2 spike glycoprotein (PDB ID: 6VSB) was used as template (Figure 3). The 3D model obtained for S-RBD of SARS-CoV-2 was validated using PROCHECK, ProSA and SAVES-Verify 3D server. Ramachandran Plot of the predicted model of S-RBD domain of spike protein by PROCHECK and SAVES-Verify 3D server suggests that 82.8% of the residues are present in the core allowed region, 15.2% in allowed region, 1.4 % in generously allowed region, and only 0.7% residues in disallowed region not part of loop involved in ACE2 receptor binding (Figure 4A). Overall, the modeled structure was good as more than 99% of the residues, after summing up, were in allowed region of Ramachandran plot.

Further validation of model was done using ProSA, where the protein folding energy obtained through it was in good agreement with the plot. The Z-score value obtained through it was −7.39 (Figure 4B). Overall quality factor evaluated by VERIFY3D was ~85%. These results suggested that the modeled S-RBD of SARS-CoV-2 is acceptable and could be further used for structure-based virtual screening. This predicted model of S-RBD of SARS-CoV-2 was used for protein-protein docking studies to identify its residues interacting with ACE2 receptor and to further screen small compounds which could block these interactions of S-RBD–ACE2 interface. The predicted homology model for S1 subunit of S-protein was submitted in PMDB database (PMDB ID: PM0082972), and the HADDOCK tool was used to identify interacting residues between receptor and S-RBD of S-protein (Figure S2).

### Receptor Binding Residues on S-RBD of SARS-CoV-2

Crystal structure of S-protein of SARS-CoV-2 (PDB ID: 6VSB), published recently, lacks residues present in the S-RBD region of SARS-CoV-2. Chimeric S-RBD of SARS-CoV-2 (PDB ID: 6VW1) has been reported, but the structure comprises majorly of SARS-CoV residues and contains only S-RBM of SARS-CoV-2. Therefore, S1-subunit of SARS-CoV-2 was modeled and used to identify molecular interactions with ACE2 receptor using HADDOCK based protein-protein docking tool. Hotspot 31 and hotspot 353 were fed as central residues on the basis of which S-RBD residues of the predicted model were docked (Figure S2).

### Identification of SARS-CoV-2 S-RBD Binding Molecules

**Figure 5:**
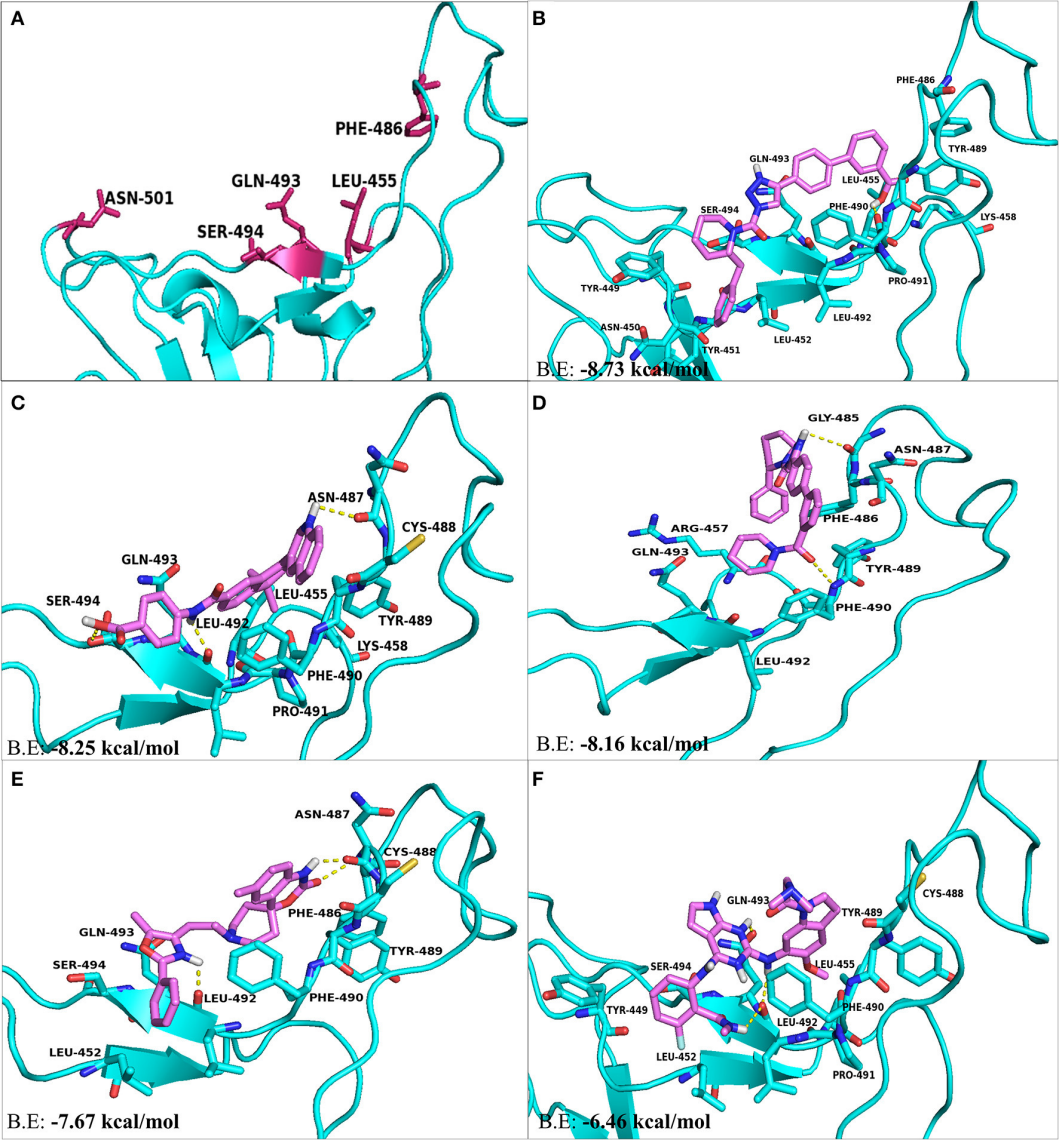
The top hit selected ligands from LOPAC library showing molecular interactions with S-RBD protein of SARS-CoV-2. **(A)** S-RBD residues responsible for interacting with ACE2 receptor. **(B)** Molecular interactions of KT203 with S-RBD. **(C)** Molecular interactions of BMS195614 with S-RBD. **(D)** Molecular interactions of KT185 with S-RBD. **(E)** Molecular interactions of RS504393 with S-RBD. **(F)** Molecular interactions of GSK1838705A with S-RBD. Blue ribbons corresponds S-RBD of spike protein of SARS-CoV-2 and violet stick model represents residues of Ligands.

The residues present at the interface region of S-RBD–ACE2 were targeted and used for structure-based screening and selection of drugs or compounds using PyRx. With respect to interface residues, AutoDock Vina based docking calculations were performed for top five molecules selected on the basis of RMSD values, binding energies and for their ability to form H-bonds and hydrophobic bonds. KT203 and BMS195614 were the first hits obtained having binding energies of −8.73 and −8.25 kcal/mol, respectively, which were more than that of KT185 (−8.16 kcal/mol) and RS504393 (−7.67 kcal/mol) (Figures 5B–E). Interestingly, the molecule RS504393 is identified to bind both ACE2 (-8.32 kcal/mol) and S-RBD (−7.67 kcal/mol) (Figure 5E). A complete list of polar and hydrophobic interactions between the five ligands and S-RBD interface are shown in Table 2.

**Table 2:** Binding energies, polar and hydrophobic interactions of selected compounds screened against S-RBD of SARS-CoV-2.

**Ligand**	**Binding energy (kcal/mol)**	**Interactions**		
		**H-Bonds**	**Bond length(Å)**	**Hydrophobic interactions**
**KT203**	**−8.73**	O3-O(Phe490)	2.85	Tyr449, Asn450, Tyr451, Leu452, Leu455, Lys458, Phe486, Tyr489, Pro491, Leu492, Gln493, Ser494
**BMS195614**	**−8.25**	N2-O(Asn487)	2.94	Leu455, Lys458, Cys488, Tyr489, Phe490, Pro491, Gln493
		N1-O(Leu492)	2.72	
		O3-OG(Ser494)	2.91	
**KT185**	**−8.16**	N4-O(Gly485) O2-N(Phe490)	2.75 2.61	Arg457, Phe486, Asn487, Tyr489, Leu492, Gln493
**RS504393**	**−7.67**	O2-N and N2-O (Asn487) N3-O(Leu492)	2.94 2.61 and 2.75	Leu452, Cys488, Phe486, Tyr489, Phe490, Gln493, Ser494
**GSK1838705A**	**−6.46**	N8-O and N4-O(Leu492)	3.14 and 2.56	Tyr449, Leu452, Leu455, Cys488, Tyr489, Phe490, Pro491, Ser494
		N2-OE1(Gln493)	2.76	

*Bold values represents names of ligands and their respective binding energies*.

### Comparison of Molecular Interactions Between S-RBD Residues and Ligands

**Figure 6:**
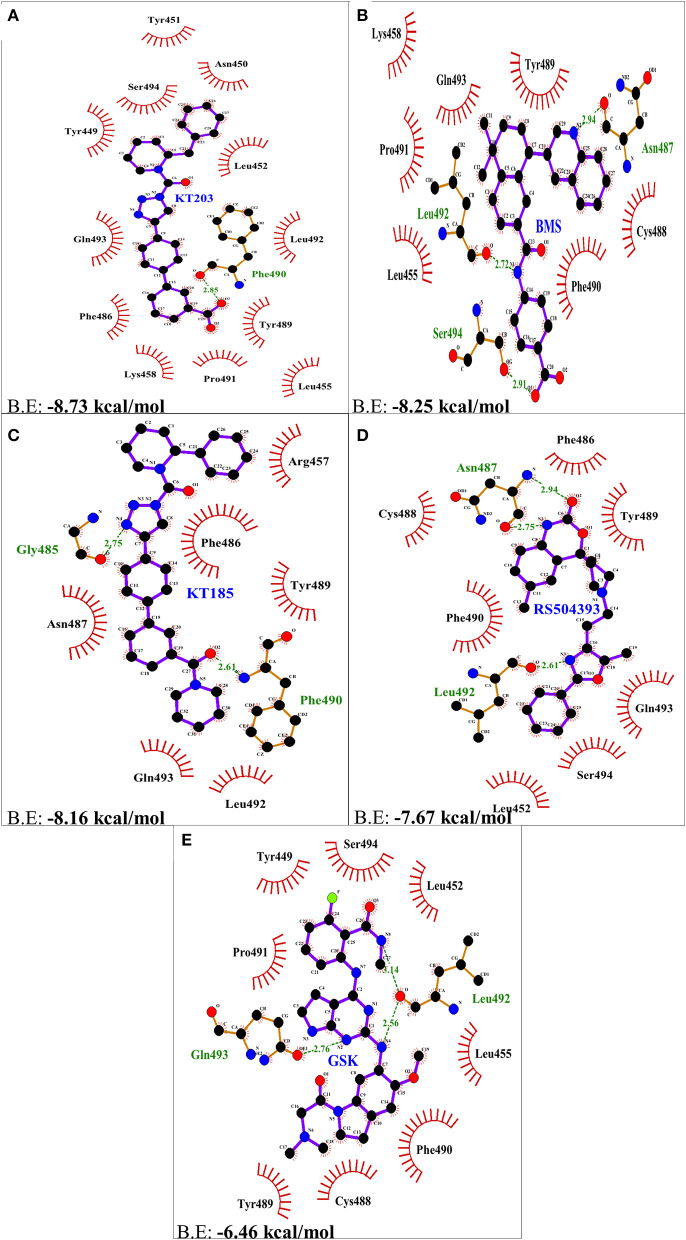
Two dimensional representation of H-bonds and hydrophobic interactions of selected compound molecules with S-RBD of S-protein using LigPlot. **(A)** KT203 **(B)** BMS195614 (BMS) **(C)** KT185 **(D)** RS5049393 **(E)** GSK1838705A (GSK). Ligands are colored and represented in purple color, H-bonds are displayed in green dotted lines, red stellations represents hydrophobic interactions and bonds of proteins are shown in brown color.

Two-dimensional plot of the molecular interaction network of the ligands with S-RBD were prepared using LigPlot, and the docked poses for each of these molecules are represented in Figure 5. The results obtained after docking calculations suggests that the S-RBD residues of SARS-CoV-2 interacting with the ligands are Leu455, Phe486, Asn487, Gln493, and Ser494. The residues Leu455, Phe486, and Gln493 of S-RBD have been reported to interact with hotspot 31, whereas residues Asn487 and Ser494 are described to interact with hotspot 353 of SARS-CoV-2 (1, 36). Out of 1280 drug molecules, KT203 and BMS195614 displayed highest binding energies of −8.73 and −8.25 kcal/mol, respectively, and interact with S-RBD residues through one and three H-bonds, respectively (Figures 5B,C). In the docked conformations, KT203 and BMS195614 displayed maximum number of hydrophobic interactions with residues responsible for recognizing both hotspot 31 and hotspot 353 (Figures 5B,C, 6A,B). Interestingly KT203 binds with Leu455, Phe486, Tyr489, Gln493, and Ser494 through hydrophobic interactions, all of which are known to be a part of virus binding motif of ACE2 receptor. Additionally, other hydrophobic interactions obtained for KT203 are Tyr449, Asn450, Tyr451, Leu452, Lys458, Pro491, and Leu492. BMS195614 interacts with Asn487 and Ser494 through H-bond and with Leu455, Lys458, Cys488, Tyr489, Phe490, Pro491, and Gln493 through hydrophobic interactions. KT185 interacts with Arg457, Phe486, Asn487, Tyr489, Leu492, and Gln493 through hydrophobic interactions (Figures 5D, 6C). Residues Gly485 and Phe490 are observed to bind with KT185 through H-bonds. RS504393 and GSK1838705A are also observed to interact with residues responsible for recognizing both hotspots (Figures 5E,F, 6D,E). RS504393 was found to be a common ligand for ACE2 receptor and S-RBD, and displayed polar interaction with Asn487 and Leu492 (Figures 5E, 6D) along with few hydrophobic interactions with Leu452, Cys488, Phe486, Tyr489, Phe490, Gln493, and Ser494. GSK1838705A displayed hydrophobic interactions with residues Tyr449, Leu452, Leu455, Cys488, Tyr489, Phe490, Pro491, and Ser494 whereas polar bonding was observed for Leu492 and Gln493. It is observed that additional H-bonds are obtained in docked complexes i.e., Gly485, Phe490, and Leu492 which seems to contribute toward stability of docked compound complexes. Cys488, Tyr489, Pro491, and Leu492 were additional important and common hydrophobic interactions observed for most of the ligands, different from ACE2 interacting residues (Table 2). To monitor the stability of protein and ligands, MD simulation was carried out. The RMSD curve converged well after 25 ns for GSK1838705A, and after 20 ns for BMS195614, KT185, KT203 and RS504393, and thereafter remained stable upto 50 ns for all ligands. Fluctuations in RMSD values were in range of 1.6 to 1.7 nm for GSK1838705A, BMS195614 and RS504393, and 2.0 to 2.1 nm for KT185. Overall, the RMSD results show that the binding of each compound to S-RBD of S-protein is stable (Figures S3A–E).

## Discussion

Understanding the virus-receptor recognition mechanism responsible for COVID-19 infection, pathogenesis and host range provides direction to develop antiviral therapy to combat and cure this global pandemic of 2020. There is no drug or antiviral treatment against SARS-CoV-2, and development of new drug molecules will take time. Moreover, WHO has already declared COVID-19 infection as a global pandemic problem, therefore repurposing drugs available for other diseases would be beneficial as these can be directly tested as anti-SARS-CoV-2 drugs and can be processed for COVID-19 trials.

Viral life cycle involves entry into the host cell after attachment to the host cell receptor, release of genetic material inside cell, synthesis of structural and non-structural proteins and genomic RNA, assembly of mature virus particles followed by budding to exit from host cell (38). RNA viruses like chikungunya virus, dengue virus, Ebola virus, SARS, MERS, Sindbis virus etc. can thus be targeted at each of these steps of their life cycle to combat infections caused by them (39). Antiviral drugs blocking entry of virus or acting on replication stages have been reported against dengue, chikungunya virus and other similar RNA viruses (40, 41). Studies suggest that targeting the capsid synthesis step could also prevent budding stage of virus (42, 43). Antiviral drugs against SARS-CoV-2 can also be identified by targeting the virus at these stages of life cycle.

Viral S-protein present on the envelope of SARS-CoV-2 is responsible for mediating interaction with ACE2 receptor present on the host cells via its RBD unit. Since this interaction is essential for SARS-CoV-2 entry into the host cell and infection, drugs targeting S-RBD–ACE2 interface protein-protein interactions could potentially inhibit the virus entry into host cell and thus, provide quick solution to control SARS-CoV-2 infections (44). Structure-based drug repurposing using high throughput virtual screening tools have been used to identify FDA approved drugs or compounds which could block interactions of SARS-CoV-2–ACE2 receptor. The results of this study of modeling of S-RBD of SARS-CoV-2, coupled with rapid screening of FDA approved LOPAC library molecules against both S-RBD and receptor ACE2, have identified potential compounds that may inhibit the virus infection.

In concordance with the results obtained after drug library screening, molecular docking studies were performed to gain insights into the binding mode and crucial molecular interactions of the selected ligands with ACE2 protein of the host cell and the S-RBD protein of SARS-CoV-2. With regards to ACE2 inhibitors, GR hydrochloride and GNF-5 interact with residues of hotspot 353 preferably and the remaining three, RS504393, TNP, and Eptifibatide acetate interact well with residues adjacent to hotspot 31 through polar as well as hydrophobic bonds. Structure-based rational drug design approach can be used to design a drug molecule combining these two ligands that will possess ability to bind both hotspot 31 and hotspot 353. KT203 and BMS195614 were predicted to be potential inhibitors against S-RBD of SARS-CoV-2 in pursuit of their high binding energies and owing to their ability to interact and block key RBD residues responsible for recognizing hotspot 31 and hotspot 353 of SARS-CoV-2 (Figures 5B,C, 6A,B). KT185, RS504393, and GSK1838705A were the other ligands obtained, and KT185 was observed to display a higher affinity toward S-RBD residue interacting with hotspot 31 by displaying interactions with Phe486 and Gln493 (Figures 5D–F). Intriguingly RS504393 was screened to be common for both S-RBD and ACE2 interface residues, with a higher affinity toward ACE2 virus binding motif.

Virtual screening of compound libraries provided some promising FDA approved drugs which are either proposed to inhibit RNA viruses by targeting entry or replication steps of their life cycle, or by providing anti-inflammatory effects. GNF-5 identified in our study, is already a reported drug that blocks coronavirus S-protein induced fusion, prior to hemifusion, by inhibiting Abl kinase (45). This drug also inhibits Dengue virus entry by its action on Abl kinase (46). Similarly TNP, identified against ACE2 is a selective inhibitor of Inositol hexakisphosphate kinase (IP6K) and Akt signaling, reported to be responsible for inhibiting MERS-CoV infection (47, 48). GR hydrochloride is an antagonist of 5-HT1B/1D serotonin receptor, and also plays a role in inhibiting entry of Ebola virus entry into host cell (49). Eptifibatide acetate protects lungs from inflammations caused by influenza virus, and has been reported as antiviral that inhibits the protease activity of Chikungunya virus capsid protein (50, 51). KT185 and KT203, inhibitors of S-RBD protein of SARS-CoV-2 are known to exert anti-inflammatory role on lungs (52, 53). GSK1838705A is known to reduce inflammations posed by infections caused by influenza virus, whereas BMS195614, another inhibitor against S-RBD is proposed to inhibit Hepatitis B virus infection (54–56). The compound RS504393, identified against both ACE2 and S-RBD, targets chemokine receptor, a mechanism by which SARS-CoV interferes with the host immune responses (57). Detailed role of screened compounds along with target sites are summarized in Table 3. Therefore, these molecules are also likely to be effective against virus by not only targeting the virus entry step but might act as anti-inflammatory drugs against cells and tissue damages caused by SARS-CoV-2 infection (50, 53).

**Table 3:** Summarized table of drugs identified against SARS-CoV-2–ACE2 receptor interface with their reported functions and role on RNA viruses.

**S. No**	**Identified compounds**	**Target in SARS-CoV-2**	**Reported function of compound**	**Inhibitory role on RNA viruses**
1	**RS504393**	SARS-CoV-2 receptor ACE2 and spike protein	Treatment of lung injury and bronchial wall thickening (58)	• Targets the chemokine receptor CCR2, responsible for intense up-regulation of chemokines, and represents a mechanism by which SARS-CoV interferes the host immune response (57).
2	**KT185**	SARS-CoV-2 spike protein	Anti-inflammatory (52)	• Inhibitor of ABHD6 receptor (53). • Inhibitor of ABHD6 receptor leads to decreased macrophage activation and is hypothesized to exert anti-inflammatory effect on brain, liver and lungs (52).
3	**KT203**			
4	**GSK1838705A**	SARS-CoV-2 spike protein	Cancer drug (54)	• Inhibitor of Insulin like growth factor-1 receptor (54). • Regulates acute inflammatory lungs injury mediated by influenza virus infection (56).
5	**BMS195614**	SARS-CoV-2 spike protein	Cancer drug (59)	• Inhibitor of Retinoic acid receptor. • Inhibits Hepatitis B virus infection by decreasing hepatocyte permissiveness, through modulation of sodium taurocholate cotransporting polypeptide (NTCP) expression (55).
6	**TNP**	SARS-CoV-2 receptor ACE2	Tyrosine kinase inhibitor	• Inhibitor of IP6K and Akt signaling pathway. • Responsible for inhibiting MERS-CoV infection by targeting Akt signaling (47, 48).
7	**GNF-5**	SARS-CoV-2 receptor ACE2	Kinase inhibitor	• Inhibits dengue virus entry and post entry step by targeting Abl kinase inhibitor (46). • Blocks coronavirus S-protein induced fusion prior to hemifusion by Abl kinase inhibition action (45).
8	**GR127935 hydrochloride hydrate**	SARS-CoV-2 receptor ACE2	Controls vasoconstriction	• Antagonist of 5-HT1B/1D serotonin receptor. • Serotonin antagonists are potent entry inhibitors of Ebola and Marburg virus (49).
9	**Eptifibatide acetate**	SARS-CoV-2 receptor ACE2	Lung injury and inflammation	• Inhibitor of glycoprotein IIb/IIIa receptor responsible for platelet aggregation (51). • Protects lungs from severe injury and inflammations induced by Influenza virus (50) • An inhibitor of capsid protease of chikungunya virus, thereby will prevent capsid synthesis during virus replication cycle (51).

## Data Availability Statement

The datasets presented in this study can be found in online repositories. The names of the repository/repositories and accession number(s) can be found in the article/Supplementary Material.

## Author Contributions

YM involved in the conceptualization, manuscript draft, and reviewing. ST involved in the conceptualization, design, analysis of data, and manuscript drafting. SC performed all computational studies, analyzed the data, and did manuscript drafting. All authors contributed to the article and approved the submitted version.

## Conflict of Interest

The authors declare that the research was conducted in the absence of any commercial or financial relationships that could be construed as a potential conflict of interest.

## References

[1] Wan Y Shang J Graham R Baric RS Li F. Receptor recognition by novel coronavirus from Wuhan: an analysis based on decade-long structural studies of SARS. J Virol. (2020) 94:e00127-20. 10.1128/JVI.00127-20 31996437 PMC7081895

[2] Malik YS Sircar S Bhat S Sharun K Dhama K Dadar M. Emerging novel coronavirus (2019-nCoV)—current scenario, evolutionary perspective based on genome analysis and recent developments. Vet Q. (2020) 40:68–76. 10.1080/01652176.2020.1727993 32036774 PMC7054940

[3] Huang C Wang Y Li X Ren L Zhao J Hu Y. Clinical features of patients infected with 2019 novel coronavirus in Wuhan, China. Lancet (London, England). (2020) 395:497–506. 10.1016/S0140-6736(20)30183-5 31986264 PMC7159299

[4] World Health Organization Situation Report 80 (2020). Available online at: https://www.who.int/docs/default-source/coronaviruse/situation-reports/20200409-sitrep-80-covid-19.pdf?sfvrsn=1b685d64_6 (accessed April 9, 2020).

[5] Liu Z Xiao X Wei X Li J Yang J Tan H. Composition and divergence of coronavirus spike proteins and host ACE2 receptors predict potential intermediate hosts of SARS-CoV-2. J Med Virol. (2020) jmv.25726. 10.1002/jmv.25726 32100877 PMC7228221

[6] World Health Organization Summary of Probable SARS Cases with Onset of Illness from 1 November 2002 to 31 July 2003 (2015). Available online at: https://www.who.int/csr/sars/country/table2004_04_21/en/ (accessed March 20, 2020).

[7] World Health Organization Middle East Respiratory Syndrome Coronavirus (MERS-CoV) - Saudi Arabia (2016). Available online at: https://www.who.int/emergencies/mers-cov/en/ (accessed March 20, 2020).

[8] Huang Q Herrmann A Fast assessment of human receptor-binding capability of 2019 novel coronavirus (2019-nCoV). bioRxiv [Preprint]. (2020). 10.1101/2020.02.01.930537

[9] Gaunt ER Hardie A Claas ECJ Simmonds P Templeton KE. Epidemiology and clinical presentations of the four human coronaviruses 229E, HKU1, NL63, and OC43 detected over 3 years using a novel multiplex real-time PCR method. J Clin Microbiol. (2010) 48:2940–7. 10.1128/JCM.00636-10 20554810 PMC2916580

[10] Wilder-Smith A Freedman DO. Isolation, quarantine, social distancing and community containment: pivotal role for old-style public health measures in the novel coronavirus (2019-nCoV) outbreak. J Travel Med. (2020) 27:taaa020. 10.1093/jtm/taaa020 32052841 PMC7107565

[11] Wu Z McGoogan JM. Characteristics of and important lessons from the coronavirus disease 2019 (COVID-19) outbreak in China: summary of a report of 72 314 cases from the Chinese Center for Disease Control and Prevention. JAMA. (2020) 323:1239–42. 10.1001/jama.2020.2648 32091533

[12] Touret F de Lamballerie X. Of chloroquine and COVID-19. Antiviral Res. (2020) 177:104762. 10.1016/j.antiviral.2020.104762 32147496 PMC7132364

[13] Elshabrawy HA Coughlin MM Baker SC Prabhakar BS. Human monoclonal antibodies against highly conserved HR1 and HR2 domains of the SARS-CoV spike protein are more broadly neutralizing. PLoS ONE. (2012) 7:e50366. 10.1371/journal.pone.0050366 23185609 PMC3503966

[14] Dhama K Sharun K Tiwari R Dadar M Malik YS Singh KP. COVID-19, an emerging coronavirus infection: advances and prospects in designing and developing vaccines, immunotherapeutics, and therapeutics. Hum Vaccin Immunother. (2020) 16:1–7. 10.1080/21645515.2020.1735227 32186952 PMC7103671

[15] Hoffmann M Kleine-Weber H Schroeder S Mü MA Drosten C Pö S. SARS-CoV-2 cell entry depends on ACE2 and TMPRSS2 and is blocked by a clinically proven protease inhibitor. Cell. (2020) 181:1–10. 10.1016/j.cell.2020.02.052 32142651 PMC7102627

[16] Wong SK Li W Moore MJ Choe H Farzan M. A 193-amino acid fragment of the SARS Coronavirus S protein efficiently binds angiotensin-converting enzyme 2. J Biol Chem. (2004) 279:3197–201. 10.1074/jbc.C300520200 14670965 PMC7982343

[17] Bonavia A Zelus BD Wentworth DE Talbot PJ Holmes K V. Identification of a receptor-binding domain of the spike glycoprotein of human coronavirus HCoV-229E. J Virol. (2003) 77:2530–8. 10.1128/JVI.77.4.2530-2538.2003 12551991 PMC141070

[18] Chan JF-W Kok K-H Zhu Z Chu H To KK-W Yuan S. Genomic characterization of the 2019 novel human-pathogenic coronavirus isolated from a patient with atypical pneumonia after visiting Wuhan. Emerg Microbes Infect. (2020) 9:221–36. 10.1080/22221751.2020.1719902 31987001 PMC7067204

[19] Andersen KG Rambaut A Lipkin WI Holmes EC Garry RF. The proximal origin of SARS-CoV-2. Nat Med. (2020) 26:450–2. 10.1038/s41591-020-0820-9 32284615 PMC7095063

[20] Wu K Li W Peng G Li F. Crystal structure of NL63 respiratory coronavirus receptor-binding domain complexed with its human receptor. Proc Natl Acad Sci USA. (2009) 106:19970–4. 10.1073/pnas.0908837106 19901337 PMC2785276

[21] Harrison C. Coronavirus puts drug repurposing on the fast track. Nat Biotechnol. (2020) 38:379–91. 10.1038/d41587-020-00003-1 32205870

[22] Li F Li W Farzan M Harrison SC. Structural biology: structure of SARS coronavirus spike receptor-binding domain complexed with receptor. Science. (2005) 309:1864–8. 10.1126/science.1116480 16166518

[23] Wu F Zhao S Yu B Chen Y-M Wang W Song Z-G. A new coronavirus associated with human respiratory disease in China. Nature. (2020) 579:265–9. 10.1038/s41586-020-2008-3 32015508 PMC7094943

[24] Dallakyan S Olson AJ. Small-molecule library screening by docking with PyRx. Methods Mol Biol. (2015) 1263:243–50. 10.1007/978-1-4939-2269-7_19 25618350

[25] O'Boyle NM Banck M James CA Morley C Vandermeersch T Hutchison GR. Open babel: an open chemical toolbox. J Cheminform. (2011) 3:33. 10.1186/1758-2946-3-33 21982300 PMC3198950

[26] Trott O Olson AJ. AutoDock Vina: improving the speed and accuracy of docking with a new scoring function, efficient optimization, and multithreading. J Comput Chem. (2010) 31:455–61. 10.1002/jcc.21334 19499576 PMC3041641

[27] Schrodinger LLC The PyMOL Molecular Graphics System, Version 1.8. Schrodinger LLC (2015)

[28] Pronk S Páll S Schulz R Larsson P Bjelkmar P Apostolov R. GROMACS 4.5: a high-throughput and highly parallel open source molecular simulation toolkit. Bioinformatics. (2013) 29:845–54. 10.1093/bioinformatics/btt055 23407358 PMC3605599

[29] Waterhouse A Bertoni M Bienert S Studer G Tauriello G Gumienny R. SWISS-MODEL: homology modelling of protein structures and complexes. Nucleic Acids Res. (2018) 46:W296–W303. 10.1093/nar/gky427 29788355 PMC6030848

[30] Dominguez C Boelens R Bonvin AMJJ. HADDOCK: a protein-protein docking approach based on biochemical or biophysical information. J Am Chem Soc. (2003) 125:1731–7. 10.1021/ja026939x 12580598

[31] Berman HM Westbrook J Feng Z Gilliland G Bhat TN Weissig H. The protein data bank. Nucleic Acids Res. (2000) 28:235–42. 10.1093/nar/28.1.235 10592235 PMC102472

[32] Coordinators NR. Database resources of the National Center for Biotechnology Information. Nucleic Acids Res. (2016) 44:D7–D19. 10.1093/nar/gkv1290 26615191 PMC4702911

[33] Laskowski RA MacArthur MW Moss DS Thornton JM PROCHECK: a program to check the stereochemical quality of protein structures. J Appl Crystallogr. (1993) 26:283–91. 10.1107/S0021889892009944

[34] Wiederstein M Sippl MJ. ProSA-web: interactive web service for the recognition of errors in three-dimensional structures of proteins. Nucleic Acids Res. (2007) 35:W407–10. 10.1093/nar/gkm290 17517781 PMC1933241

[35] Eisenberg D Lüthy R Bowie JU. VERIFY3D: Assessment of protein models with three-dimensional profiles. Methods Enzymol. (1997) 277:396–404. 10.1016/S0076-6879(97)77022-8 9379925

[36] Shang J Ye G Shi K Wan Y Luo C Aihara H. Structural basis for receptor recognition by the novel coronavirus from Wuhan. (2020) 1–19. 10.21203/rs.2.24749/v131996437

[37] Yan R Zhang Y Li Y Xia L Zhou Q. Structure of dimeric full-length human ACE2 in complex with B 0 AT1. bioRxiv [Preprint]. (2020) 10.1101/2020.02.17.95184832132184

[38] Dimitrov DS. Virus entry: molecular mechanisms and biomedical applications. Nat Rev Microbiol. (2004) 2:109–22. 10.1038/nrmicro817 15043007 PMC7097642

[39] Kaur R Neetu Mudgal R Jose J Kumar P Tomar S. Glycan-dependent chikungunya viral infection divulged by antiviral activity of NAG specific chi-like lectin. Virology. (2019) 526:91–8. 10.1016/j.virol.2018.10.009 30388630

[40] Singh H Mudgal R Narwal M Kaur R Singh VA Malik A. Chikungunya virus inhibition by peptidomimetic inhibitors targeting virus-specific cysteine protease. Biochimie. (2018) 149:51–61. 10.1016/j.biochi.2018.04.004 29635044

[41] Mudgal R Mahajan S Tomar S. Inhibition of Chikungunya virus by an adenosine analog targeting the SAM-dependent nsP1 methyltransferase. FEBS Lett. (2020) 594:678–94. 10.1002/1873-3468.13642 31623018 PMC7164056

[42] Sharma R Kesari P Kumar P Tomar S. Structure-function insights into chikungunya virus capsid protein: small molecules targeting capsid hydrophobic pocket. Virology. (2018) 515:223–34. 10.1016/j.virol.2017.12.020 29306785

[43] Sharma R Fatma B Saha A Bajpai S Sistla S Dash PK. Inhibition of chikungunya virus by picolinate that targets viral capsid protein. Virology. (2016) 498:265–76. 10.1016/j.virol.2016.08.029 27614702

[44] Adedeji AO Severson W Jonsson C Singh K Weiss SR Sarafianos SG. Novel inhibitors of severe acute respiratory syndrome coronavirus entry that act by three distinct mechanisms. J Virol. (2013) 87:8017–28. 10.1128/JVI.00998-13 23678171 PMC3700180

[45] Sisk JM Frieman MB Machamer CE. Coronavirus S protein-induced fusion is blocked prior to hemifusion by Abl kinase inhibitors. J Gen Virol. (2018) 99:619–30. 10.1099/jgv.0.001047 29557770 PMC6537626

[46] Clark MJ Miduturu C Schmidt AG Zhu X Pitts JD Wang J. GNF-2 inhibits dengue virus by targeting Abl kinases and the viral e protein. Cell Chem Biol. (2016) 23:443–52. 10.1016/j.chembiol.2016.03.010 27105280 PMC4865888

[47] Chakraborty A Koldobskiy MA Bello NT Maxwell M Potter JJ Juluri KR. Inositol pyrophosphates inhibit akt signaling, thereby regulating insulin sensitivity and weight gain. Cell. (2010) 143:897–910. 10.1016/j.cell.2010.11.032 21145457 PMC3052691

[48] Kindrachuk J Ork B Hart BJ Mazur S Holbrook MR Frieman MB. Antiviral potential of ERK/MAPK and PI3K/AKT/mTOR signaling modulation for middle east respiratory syndrome coronavirus infection as identified by temporal kinome analysis. Antimicrob Agents Chemother. (2015) 59:1088–99. 10.1128/AAC.03659-14 25487801 PMC4335870

[49] Cheng H Lear-Rooney CM Johansen L Varhegyi E Chen ZW Olinger GG. Inhibition of ebola and marburg virus entry by G protein-coupled receptor antagonists. J Virol. (2015) 89:9932–8. 10.1128/JVI.01337-15 26202243 PMC4577884

[50] Lê VB Schneider JG Boergeling Y Berri F Ducatez M Guerin JL. Platelet activation and aggregation promote lung inflammation and influenza virus pathogenesis. Am J Respir Crit Care Med. (2015) 191:804–19. 10.1164/rccm.201406-1031OC 25664391

[51] Fatma B Kumar R Singh VA Nehul S Sharma R Kesari P. Alphavirus capsid protease inhibitors as potential antiviral agents for Chikungunya infection. Antiviral Res. (2020) 179:104808. 10.1016/j.antiviral.2020.104808 32380148

[52] Bottemanne P Paquot A Ameraoui H Alhouayek M Muccioli GG. The α/β-hydrolase domain 6 inhibitor WWL70 decreases endotoxin-induced lung inflammation in mice, potential contribution of 2-arachidonoylglycerol, and lysoglycerophospholipids. FASEB J. (2019) 33:7635–46. 10.1096/fj.201802259R 30896979

[53] Hsu K-L Tsuboi K Chang JW Whitby LR Speers AE Pugh H. Discovery and optimization of piperidyl-1,2,3-triazole ureas as potent, selective, and *in vivo*-Active Inhibitors of α/β-Hydrolase domain Containing 6 (ABHD6). J Med Chem. (2013) 56:8270–9. 10.1021/jm400899c 24152295 PMC3987869

[54] Sabbatini P Korenchuk S Rowand JL Groy A Liu Q Leperi D. GSK1838705A inhibits the insulin-like growth factor-1 receptor and anaplastic lymphoma kinase and shows antitumor activity in experimental models of human cancers. Mol Cancer Ther. (2009) 8:2811–20. 10.1158/1535-7163.MCT-09-0423 19825801

[55] Tsukuda S Watashi K Iwamoto M Suzuki R Aizaki H Okada M. Dysregulation of retinoic acid receptor diminishes hepatocyte permissiveness to hepatitis B virus infection through modulation of sodium taurocholate cotransporting polypeptide (NTCP) expression. J Biol Chem. (2015) 290:5673–84. 10.1074/jbc.M114.602540 25550158 PMC4342479

[56] Li G Zhou L Zhang C Shi Y Dong D Bai M. Insulin-like growth factor 1 regulates acute inflammatory lung injury mediated by influenza virus infection. Front Microbiol. (2019) 10:2541. 10.3389/fmicb.2019.02541 31849847 PMC6887893

[57] Kwiatkowski K Piotrowska A Rojewska E Makuch W Mika J. The RS504393 influences the level of nociceptive factors and enhances opioid analgesic potency in neuropathic rats. J Neuroimmune Pharmacol. (2017) 12:402–19. 10.1007/s11481-017-9729-6 28337574 PMC5527054

[58] Yang D Tong L Wang D Wang Y Wang X Bai C. Roles of CC chemokine receptors (CCRs) on lipopolysaccharide-induced acute lung injury. Respir Physiol Neurobiol. (2010) 170:253–9. 10.1016/j.resp.2010.02.002 20152938

[59] Hammond LA Krinks CH Van Durham J Tomkins SE Burnett RD Jones EL. Antagonists of retinoic acid receptors (RARs) are potent growth inhibitors of prostate carcinoma cells. Br J Cancer. (2001) 85:453–62. 10.1054/bjoc.2001.1939 11487280 PMC2364081

[60] Choudhary S Malik YS Tomar S Identification of SARS-CoV-2 cell entry inhibitors by drug repurposing using in silico structure-based virtual screening approach. chemRxiv [Preprint]. (2020). 10.26434/chemrxiv.12005988 PMC7365927 32754161

