# Supplementary material for: Identification of SARS-CoV-2 Cell Entry Inhibitors by Drug Repurposing Using in silico Structure-Based Virtual Screening Approach
Source: Front Immunol. 2020 Jul 10;11:1664. doi: 10.3389/fimmu.2020.01664 (PMC7365927; doi:10.3389/fimmu.2020.01664)
Supplement: Supplementary file 1 [file Data_Sheet_1.docx]

**Supplementary Files**


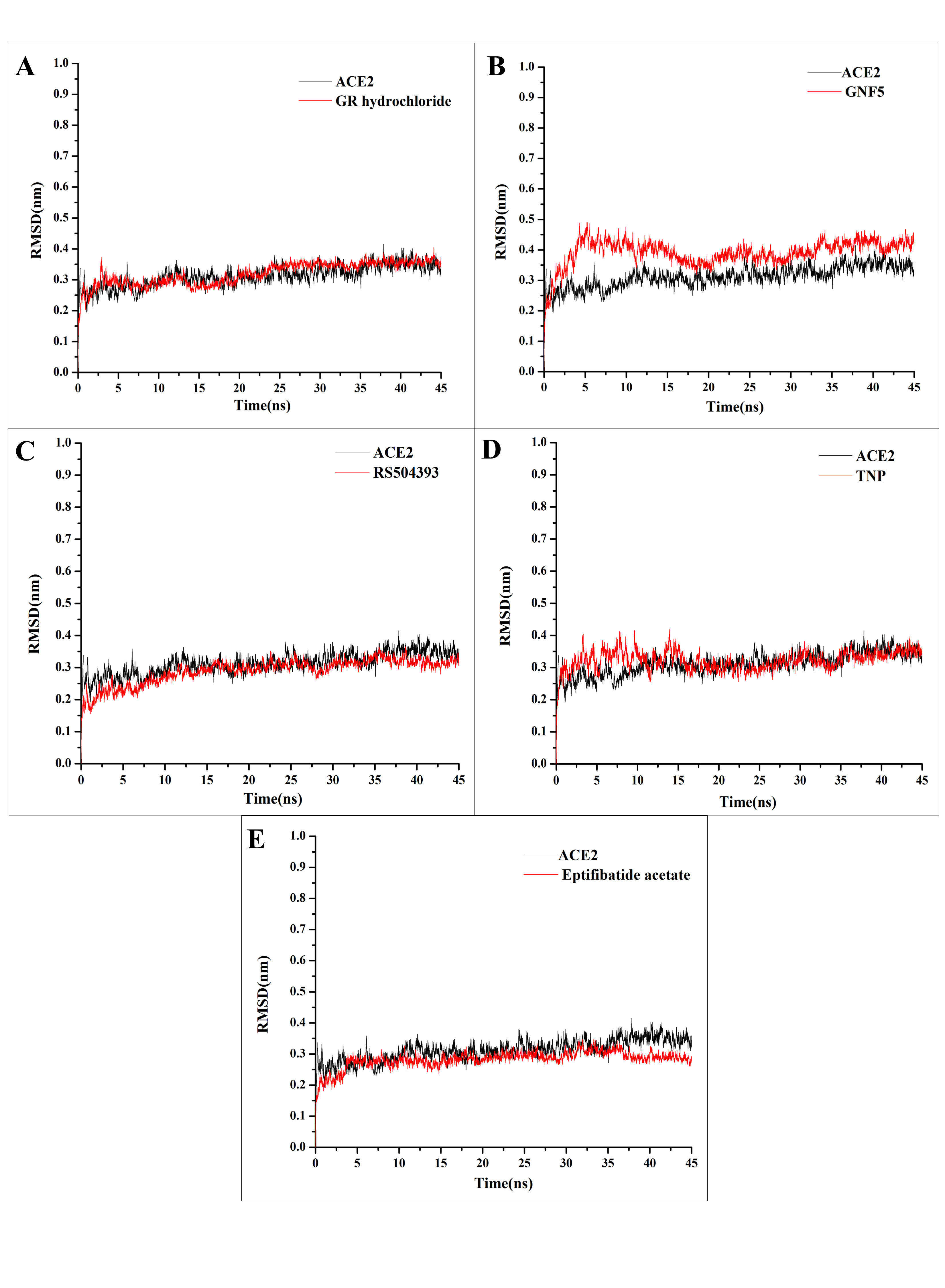


**Figure S1 |** RMSD values for native ACE2 protein and protein-ligand complexes. The RMSD plot for 45ns simulation run for protein-inhibitor complexes **(A)** ACE2-GR hydrochloride complex **(B)** GNF-5 **(C)** RS504393 **(D)** TNP and **(E)** Eptifibatide acetate. RMSD plot for ACE2 is depicted in Black color, and ACE2-inhibitor complexes are represented in Red color.

**
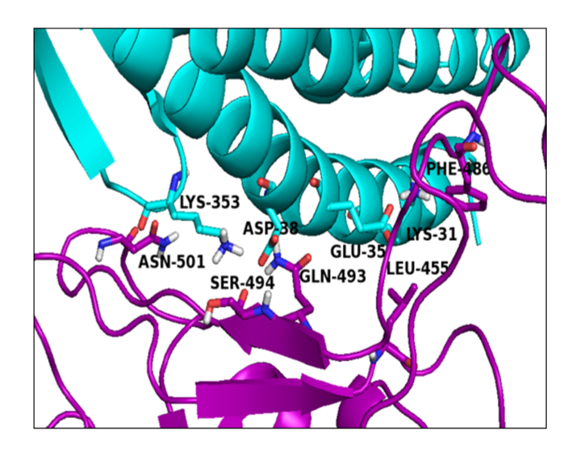
**

**Figure S2 |** The S-RBD of S-protein and ACE2 receptor interface showing molecular interactions of residues involved in binding of SARS-CoV-2 virus to the receptor


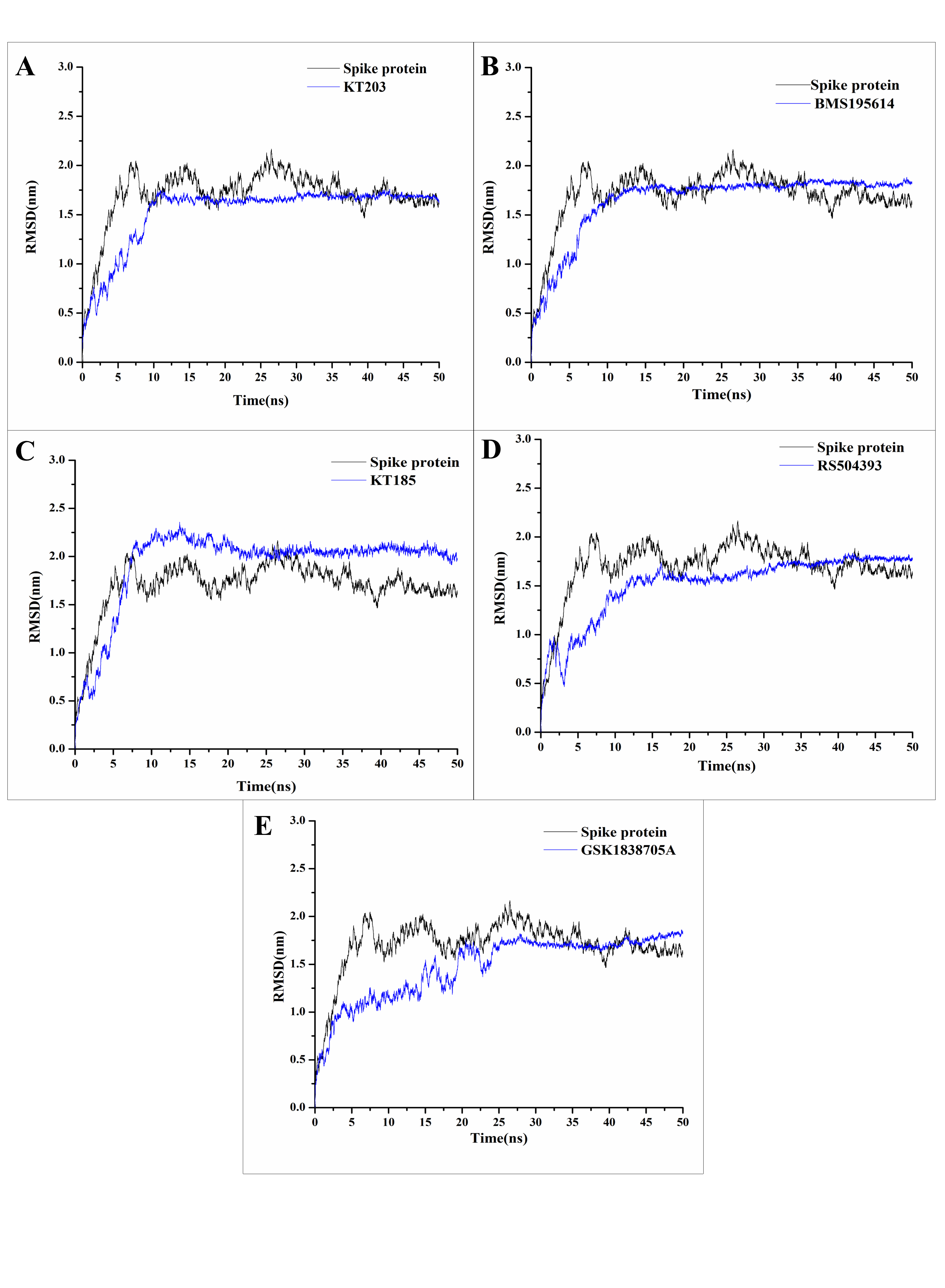


**Figure S3 |** RMSD values for native S1 subunit of S-protein and protein-ligand complexes. The RMSD plot for 50ns simulation run for protein-inhibitor complexes **(A)** KT203 **(B)** BMS195614**(C)** KT185 **(D)** RS504393 and **(E)** GSK1838705A. RMSD plot for S-protein is depicted in black color, and protein-inhibitor complexes are represented in blue color.
